# The impact of social motivation on the other-race effect under high and low social status

**DOI:** 10.1038/s41598-022-24333-z

**Published:** 2022-11-27

**Authors:** Gaixia Fan, Yuetan Wang, Yonglei Yue, Jin Lei, Peng Zhang, Xiaobin Ding

**Affiliations:** 1 School of Education, Northwest Normal University, Lanzhou, China; 2 School of Psychology, Northwest Normal University, 967 Anning East Road, Lanzhou, 730070 China; 3 Key Laboratory of Behavioral and Mental Health of Gansu Province, Lanzhou, Gansu China

**Keywords:** Psychology, Human behaviour

## Abstract

The other-race effect refers to the phenomenon in which the chance of individuals misidentifying faces from other races more than their own race is significantly higher. This study explored the effect of motivation on the other-race effect by manipulating the social status of faces. The results showed that: (1) compared to other-race faces with low social status, when individuals' perceptions of the social status of other-race faces increased, individuals' recognition scores for high social status other-race faces increased, and the other-race effect disappeared, and (2) when individuals' perceptions of the social status of other-race faces decreased, there was no significant difference in individuals' recognition scores of other-race faces, of either high or low social status. These findings suggest that motivation has a significant impact on the other-race effect.

## Introduction

The other-race effect (ORE), a phenomenon in which the chance of individuals misidentifying other-race faces is significantly higher than misidentifying own-race faces^1,2^, is also known as the own-race bias or the cross-race effect. Misidentifying others, especially members of different races, can cause social difficulties for individuals^3^ and affect judicial justice^4^ and ethnic relations^5^. There has always been extensive attention from researchers regarding the causes of the ORE^6–8^. Research on this issue can help enhance understanding of the ORE, reduce related social problems, and promote interpersonal harmony.

Previous research on the causes of the ORE has mainly focused on areas such as contact experience or social classifications. Theories centered on contact experience believe that individuals have developed more perceptual experience of distinguishing and recognizing faces of their own race through long-term contact with members of their own race, resulting in recognition advantages for own-race faces^7^. Theories centered on social classifications suggest that the division of in-group and out-group members from different racial groups enables individuals to adopt a more advantageous " conformational processing" strategy regarding faces of their own race (in-group members), resulting in recognition advantages for own-race faces^9,10^. These studies hypothesize that cognitive processes are an essential factor influencing the ORE. Besides perceptual experience account, Hugenberg's model also highlights that social-cognitive factors play an important role in shaping the ORE^7,11,12^. Dalmaso et al. (2012) research showed high-status faces have a more gaze than low-status faces, which has nothing to do with the specific characteristics of the face. Social status deeply shapes social interactions, and that humans are particularly sensitive to social hierarchies. Indeed, people preferentially allocate attentional resources to high-status individuals. In this regard, it has recently been demonstrated that people tend to gaze at high-status individuals more often and for longer than at low-status individuals^13^, and that high-status faces are better encoded in memory and processed more holistically^14^. Based on the idea that high-status individuals could be considered as more relevant sources of information when compared with low-status individual.

In addition, the motivation-focused Categorization-Individuation Model (CIM) suggests that the ORE arises due to the intrinsic motivation of perceivers' category processing of out-group members and individualized processing of in-group members^12,15,16^. Category processing is the process of assigning social members to a particular social category. It is the processing of information about the standard dimensions (category features) of category members (e.g., race), which can lead to lower recognition scores due to the high similarity in category features of faces of members of the same category and the tendency to confuse them during reidentification^17,18^. In contrast, individuation is the process of distinguishing, in detail, the identity information of each category member, and it is a diagnostic process of category member identity information and thus higher recognition scores^19,20^. Those other-race faces that are relevant to the perceiver and meet some needs of the perceiver are prone to motivate the perceiver to individualize and process other-race faces. When perceivers are motivated to individualize the processing of other-race faces, they improve the individual recognition performance of other-race faces, thus attenuating the ORE. In addition, the model argues that merely increasing contact with members of the other-race group, without the motivation to individualize processing of other-race faces, does not attenuate the ORE of face recognition^21–23^.

Kawakami et al. elicited the motivation to individualize participants' processing of other-race faces by asking White participants to look closely at and memorize Black faces through a guiding phrase and found that participants' recognition scores for other-race faces increased compared to the group of participants who did not use the guiding phrase^15^. Baldwin et al. elicited motivation to individualize participants' processing of other-race faces by increasing the perceiver's connection to members of the opposite race and found that compared to those who were told to be partnered with White individuals, participants' performance in recognizing Black faces was increased significantly when they were partnered with Black individuals^24^. In summary, the ORE seems to be malleable under certain motivational conditions. In this study, we further focus on the generation and influencing factors of the alien effect of faces from the perspective of social motivation.

Members with high social status may be respected, admired, and obeyed by others in their social environment^25^, which could be used as a motivator. Many studies have found that members of society with higher social status tend to receive more respect and admiration from others and that humans, as a social group, have an intrinsic need to be respected by others. Therefore, individuals will satisfy their intrinsic needs through various behaviors (e.g., choosing jobs or environments with higher social status), thus giving rise to an internal motivation that leads them to pursue high social status^26,27^. According to the explanation of evolutionary psychology, individuals are internally motivated to pursue high social status because it provides individuals with more resources for survival and development^28^. Researchers have also used eye-movement techniques to explore the internal mechanisms by which social status triggers social motivation. In Dalmaso, Pavan, Castelli, and Galfano, participants were asked to view 16 pictures of faces after reading statements that indicated the high or low social status of the individuals in the pictures^29^. The results showed that participants spent significantly more time looking at the high social status faces. This may be because high social status faces can motivate individuals to aspire to high social status. Therefore, the individuals would allocate more gaze time or cognitive resources to the higher social status faces, thereby increasing their expectation of being respected and obeyed by others. In summary, social status, as an intrinsic need of individuals, generates motivation.

This study aimed to examine the relationship between motivation and the ORE by manipulating social status. Study 1 examined the relationship between motivation and the ORE by increasing the participants' perceptions of the social status of other-race faces by presenting face images in combination with labels. According to Hugenberg et al. manipulations of social status can lead to individuals re-perceiving other-race faces based on social status, thereby increasing participants' motivation to individualize and process other-race faces^12^. Conversely, if participants' perceptions of the social status of other racial faces are attenuated, would participants' recognition advantage in identifying other racial faces with high social status be lost due to diminished motivation? Based on research on emotional priming on the individual attentional bias, it is known that emotional picture priming affects participants' attentional bias. If the emotional priming picture is more congruent with the target stimulus (picture, text), participants tend to gaze at and allocate more cognitive resources to the target stimulus that is more congruent with the priming emotional picture^30^. Thus, Study 2 also attenuated participants' perceptions of the social status of other-race faces through this manipulation, which attenuated participants' motivation and cognitive resources to individualize and process other-race faces. Specifically, scenes representing high and low social status were selected as priming stimuli to attenuate the participants' perception of the social status of the pictures of foreign faces. The scenes were selected from the Chinese scenes, and then a pair of Chinese and other-race faces matching the social status of the scenes was presented to allow the participants to allocate more attention and cognitive resources to the native faces.

We hypothesized that, firstly, if motivation is necessary to produce the ORE, individuals' recognition scores for high social status other-race faces would significantly increase when individuals' perceptions of the social status of other-race faces are triggered, thereby attenuating the ORE of face recognition. Secondly, no significant differences in individuals' recognition scores for other-race faces would occur when the cognitive resources required for individuals' perception of the social status of other-race faces are more occupied by faces of their race that are consistent with the priming stimuli.

## Study 1

### Method

#### Participants

G*Power 3.1.9.245 was used to estimate the planned sample size (*α* = 0.05, (1 − *β*) = 0.95), and at least 36 participants were required for calculation. Forty-six college students (23 females, 23 males) aged 20–24 years (21.54 ± 2.89) with normal or corrected vision correctly understood the experimental task and signed an informed consent form before the start of the experiment. The research was conducted under the Declaration of Helsinki guidelines and was approved by the Ethics Committee of Northwest Normal University.

#### Study design

The current study used a 2 (racial faces: other-race faces; own-race faces) × 2 (social status: high social status occupation labels—doctor, university professor, military officer, CEO; low social status occupation labels—civilian worker, courier, waiter, cleaner) within-participant experimental design.

#### Stimuli

Face pictures: there were 64 pictures in total from the PAL/CAL database (Minear and Park 2004) (32 own-race faces, 32 other-race faces, 50 male faces, and 50 female faces). Occupation labeling: 12 participants who did not participate in the experiment were asked to classify the social status for eight occupations (CEO, doctor, military officer, university professor, courier, restaurant waiter, janitor, and civilian worker) as high and low. As expected, the occupation labels with high social status were usually classified in the higher social status category, while those with low social status were usually classified in the lower social status category. Finally, these occupational labels were added below the images of faces of different races.

#### Procedures

Participants perform experiments separately in a quiet room in the laboratory. Before the experiment began, they were informed that the whole experiment consisted of two phases: learning and recognition. The main task of the learning phase was to try to learn and memorize the 32 faces in the center of the screen, which were collected from members of society from different countries and different occupational fields. The recognition phase was simply to determine whether the faces appearing in the center of the screen in sequence had appeared during the learning phase. The specific experimental procedures were as follows: a black gaze point was first presented at the center of the screen for 800 ms, followed by 2000 ms of pictures of faces with different occupational labels below, all pictures were presented only once, and the learning phase ended. Immediately after the participants completed an irrelevant task for about two minutes, they entered the recognition phase, in which they were asked to respond whether they had seen the 64 faces from different countries and occupations presented in the center of the screen (half of them were old and half were new). They pressed the "F" key if they had seen them and the "J" key if they had not. The picture will disappear only after a response. The duration was the time it took for the participants to finish responding. All labels and faces were presented randomly in the experiment.

### Results

This study uses the discrimination index (*d′*) of signal detection theory to analyze the discrimination ability of participants in recognizing faces of different races and social statuses. The *d′* is a standard parameter used to measure the ability of participants to distinguish old and new faces. It can be obtained from the hit rate (P ("Yes"/Signal)) and the false alarms rate (P ("Yes"/Noise)), and the Z_(H)_ and Z_(F)_ corresponding to the two probabilities can be found using the POZ conversion table, which is expressed by the formula:

$$d^{\prime} \, = {\text{ Z}}_{{({\text{H}})}} - {\text{ Z}}_{{({\text{F}})}} .$$

The larger *d′* means the more substantial discriminative power; the smaller *d′* means weaker discriminative power. The discriminative power (*d′*) is an essential indicator of the ability to distinguish between old and new faces, and the reporting criterion *C* indicates the stringency of the participant's judgment standard, which is often calculated by the formula: *Z*_(H)_ + *Z*_(F)_/2.

**Figure 1:**
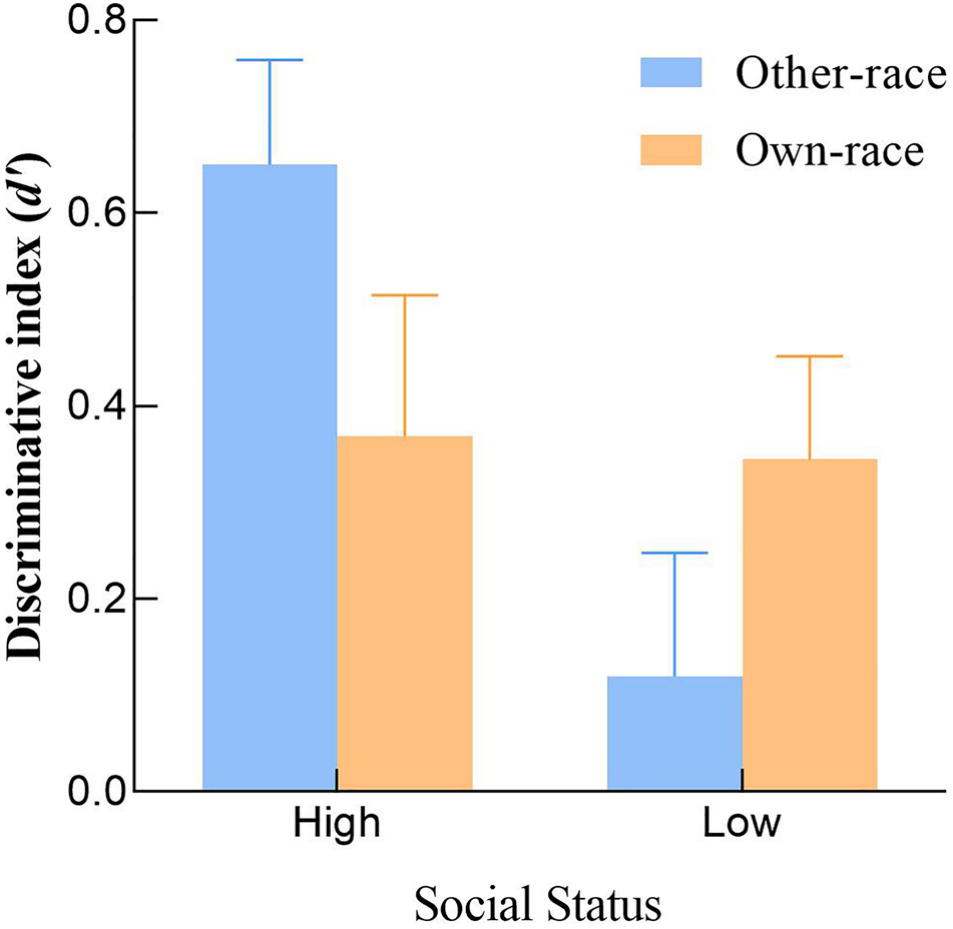
Histogram of the interaction between racial faces and the mean discriminative index (*d′)* of social status in Study 1. Error bars: ± SEM.

Participants' hit rate, false alarms rate, discriminative index *d′*, and reporting criteria *C*, for pictures of faces of different racial ethnicities and social statuses during the recognition phase, are shown in Table 1. Censoring was performed using *d′* as the indicator, where one participant had all 0 s for the discrimination indicator (*d′*), so one participant's data was removed. Based on the data of the remaining 45 participants, a repeated-measures ANOVA of 2 (racial faces: other-race faces, own-race face) × 2 (occupation labels: high social status occupation label, low social status occupation label) was conducted, with the discriminative index *d′* of the participants' recognition of different types of faces as the dependent variable (see Fig. 1). Results found that participants' discriminative index *d′* for faces with high social status occupations was significantly better than that for faces with low social status occupations, *F* (1, 44) = 4.21, *p* < 0.05, *η*_*p*_^2^ = 0.084. Participants' discriminative power for faces of different races did not differ significantly, *F* (1, 44) = 0.12, *p* , *p* > 0.05, *η*_*p*_^2^ = 0.001. The interaction between racial faces and social status was significant, *F* (1, 44) = 4.32, *p* < 0.05, *η*_*p*_^2^ = 0.087. A subsequent simple effects analysis of the interaction found that when it was a other-race face, participants' discrimination index *d′* for faces with high social status occupations was significantly better than that for faces with low social status occupations, *F* (1, 44) = 7.32, *p* < 0.01, *η*_*p*_^2^ = 0.186. When it was an own-race face, there was no significant difference between participants' discriminatory index for faces with high social status occupations *d′* and the discriminative index *d′* for faces with low social status occupations, *F* (1, 44) = 0.12,* p* > 0.05, *η*_*p*_^2^ < 0.001. When the target faces were of high social status, there was no significant difference in d′ between own-race and other-race faces (*F* = 2.774, *p* = 0.103, *η*_*p*_^2^ = 0.058). Similarly, when the target faces were of low social status, there was no significant difference in d′ between own-race and other-race faces (*F* = 2.232, *p* = 0.142, *η*_*p*_^2^ = 0.048). The above results indicate that participants did not show the recognition advantage of faces with high social status in their own racial group but showed a significant recognition advantage for high social status faces when recognizing faces of different races with different social statuses.

**Table 1:** Average hit rate, misrepresentation rate, d′, and C in Study 1.

Category	High social status				Low social status			
	Hit rate	False alarms rate	*d′*	*C*	Hit rate	False alarms rate	*d′*	*C*
Other-race	0.80 (0.82)	0.15 (0.58)	0.65 (0.73)	0.17 (0.61)	0.30 (0.8)	0.18 (0.56)	0.12 (0.86)	0.24 (0.54)
Own-race	0.45 (0.88)	0.10 (0.71)	0.36 (0.98)	0.28 (0.64)	0.48 (0.9)	0.13 (0.71)	0.34 (0.71)	0.30 (0.73)

The numbers outside the brackets in the table are the means, and the numbers inside the brackets are the standard deviations.

Reporting criteria *C*: The reporting criteria of the participants under each condition were relatively stringent (above "0"). However, neither the main effect of racial faces (*F* (1, 44) = 0.641, *p* = 0.428, *η*_*p*_^2^ = 0.014) nor social status (*F* (1, 44) = 0.313, *p* = 0.579, *η*_*p*_^2^ = 0.007) was significant, and the interaction (*F* (1, 44) = 0.072, *p* = 0.789, *η*_*p*_^2^ = 0.002) was not significant.

### Discussion

Based on the relationship between occupational status and social status, Study 1 explored the effect of motivation on the other-face effect by manipulating the social status of faces through occupational labels. The results showed that individuals' recognition scores for other-race faces with high social status occupation labels significantly improved, and the ORE diminished, demonstrating the impact of motivation on the ORE. However, the social status manipulation did not result in significantly better individual recognition scores for own-race faces with high social status than those with low social status. In contrast to the results of the current study, Shriver et al. found that when associated with low economic status cues, native faces were identified as out-group members, which would reduce the recognition of native faces but not the ORE^31^. This could be possible because Shriver et al.'s study embedded faces directly into background pictures representing different socioeconomic statuses, whereas the present study used textual categorical labels and presented a different choice of stimulus paradigm. The current study prefers to mobilize participants' intrinsic motivation, examine the influence of psychological factors in participants' recognition of interracial faces, and detect the interracial effect on social status differences from the perspective of intrinsic motivation. Also, the fact that the participants recruited in the two studies had East–West cultural differences may be a potential reason.

Social status and the other-race effect of faces belong to the category of social classification. However, there may be differences in the effects of social status and ethnicity on face recognition, which the hierarchical nature of social classification may cause. Social status did not affect the recognition of their own-race faces, but it could affect the recognition of other-race faces. Other-race faces seem to receive more attention than own-race faces, which induces the possible existence of the social class effect. We speculate that face ethnicity and social status belong to different levels of social classification. However, future cross-cultural studies are needed to further elucidate this question.

## Study 2

Study 1 showed that participants' recognition scores for other-race faces with high social status increased significantly, and the ORE disappeared. However, does attenuating individuals' perceptions of the social status of other-race faces decrease participants' recognition scores for other-race faces with high social status, thereby increasing the ORE? Study 2 further examined the relationship between motivation and the ORE by presenting the priming stimulus followed by paired faces. In such a way, participants would allocate more cognitive resources to their own-race faces, which is consistent with the priming stimulus, and their perception of the social status of other-race faces would be attenuated^29^. We hypothesized that when participants' cognitive resources were manipulated, recognition scores for faces of their own race would increase, and recognition of faces of other races would decrease for both high and low social status faces, meaning the ORE would increase.

### Method

#### Participants

G*Power 3.1.9.245 was used to estimate the planned sample size (α = 0.05, (1 − β) = 0.95), and at least 23 participants were required for calculation. Forty-four university students (22 females, 22 males) aged 20–24 years(M ± SD = 21.32 ± 2.61) with normal or corrected vision understood the experimental task and signed an informed consent form before starting the experiment.

#### Study design

The current study used a 2 (starter stimulus: high social status scene pictures/low social status scene pictures) × 2 (racial faces: other-race faces; own-race faces) × 2 (social status: high social status; low social status occupation labels) within-participant experimental design.

#### Study materials

The face pictures and occupation labels were identical to those in Study 1, except for the eight starter pictures. The eight starter stimuli were scene pictures matching their occupational identities. All these scene pictures were selected from a Chinese context. For example, the starter stimulus picture of a CEO's face was the picture of a CEO on the Hurun Top 100 Rich List. Before we started the experiment, we asked 12 subjects to rate the social status of eight occupations. In addition, we also assessed the occupational typicality and familiarity of eight occupations in order to ensure that the subjects understood the occupations being evaluated. Likert 9-point scale was used for the above evaluation. From the evaluation results, we selected the occupations with the highest social status scores (CEO) and lowest social status scores (cleaner) as the prime materials for the formal experiment. We found a significant difference in social status scores between CEOs (8.38 ± 0.39) and cleaners (1.71 ± 0.49, *t* = 40.97, *df* = 28, *p* < 0.001).

#### Study procedures

To correctly distinguish the social status of different faces, the current study uses the background color for this purpose. The background color represents the social status of the face members. The members in the yellow background have higher social status, higher social prestige, and some special power in the society, while the members in the green background are usually at the bottom of the society, have lower salaries, and have fewer decent jobs. Your task is to try to remember and distinguish the differences between the faces, especially those appearing in yellow. To balance the effect of the background color on the recall performance, each face picture is presented in green or yellow.

**Figure 2:**
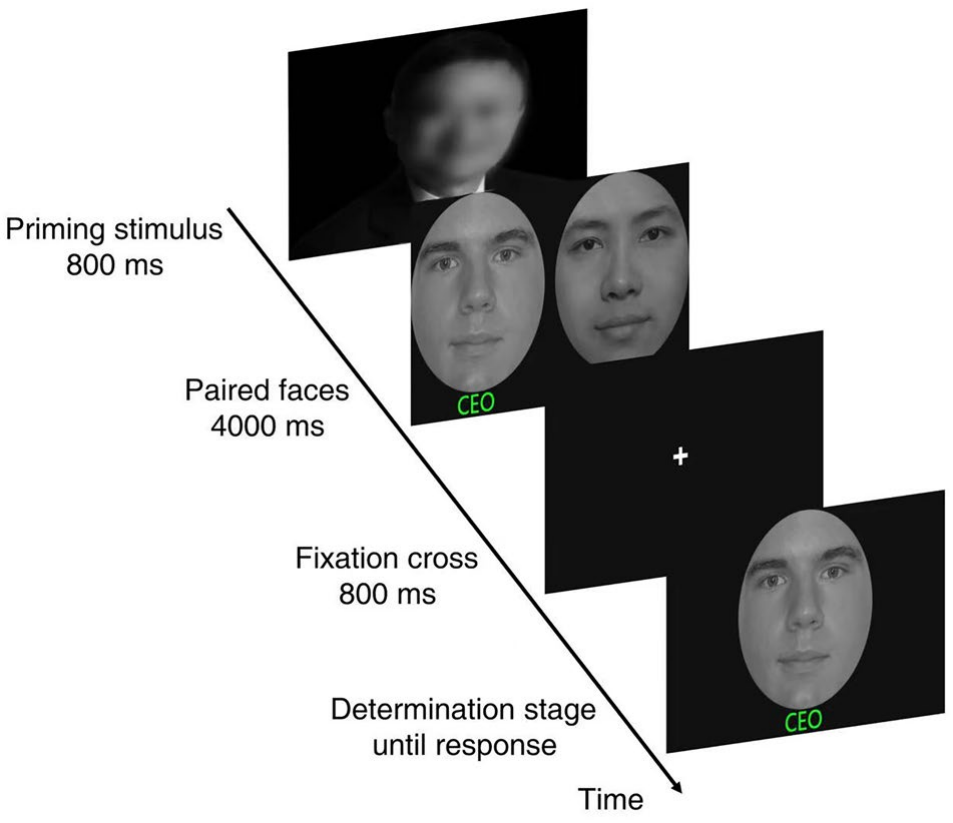
Flow chart of the experiment in Study 2.

The whole experiment includes two stages: learning and recognition. In the learning stage, participants need to memorize 32 faces in the center of the screen from members of society in different countries and professions. We presented participants with 8 prime faces of different social status, including 4 high-status faces and 4 low-status faces. The faces of high socioeconomic status are well-known company CEOs in China, such as Jack Ma. The low socioeconomic status faces are in real-life photos of cleaners. To avoid possible order effects, the order in which priming stimuli were presented was balanced between participants of different social status. We presented prime stimuli for 800 ms in the center of the screen, indicating high/low social status. Then followed the target stimuli for 4000 ms, which consisted of two other-race and own-race faces of the same professional identity (see Fig. 2). The present placement of other-race and own-race faces in paired stimuli was not fixed. At the end of the learning stage, participants completed unrelated tasks for about two minutes. In the recognition stage, participants were asked to judge whether the face shown on the screen had been presented in the learning stage. If they had seen this face, they need to press the "J" key; if this face had not seen, they need to press the "F" key.

### Results

**Figure 3:**
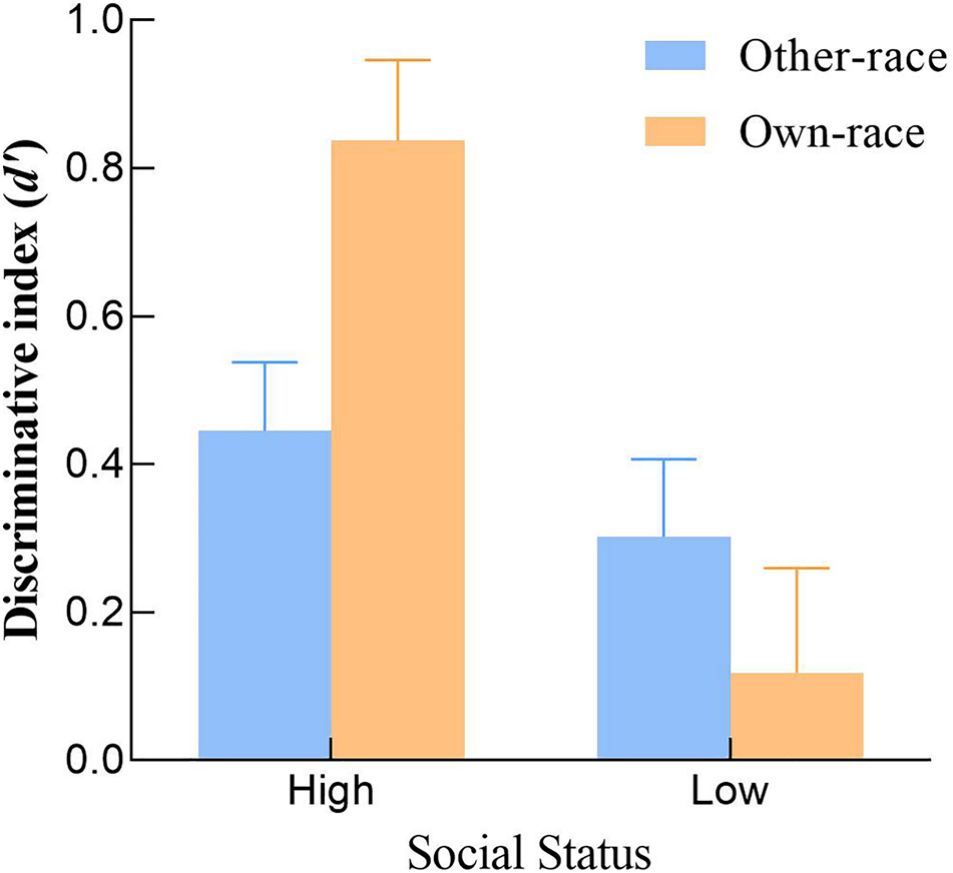
Histogram of the interaction between racial faces and the mean discriminative index (*d′)* of social status in Study 2. Error bars: ± SEM.

Participants' hit rate, false alarms rate, discriminative index *d′,* and reporting criteria *C* for pictures of faces of different races and social statuses during the recognition phase are shown in Table 2. Discriminative index (*d′*): using the discrimination indicator *d′* for participants' recognition of different types of faces as the dependent variable (see Fig. 3), a 2 (face race: other-race face, native face) × 2 (social status: high social status occupation label, low social status occupation label) repeated measures ANOVA was conducted. Results showed that participants' discriminative index *d′* for faces with high social status occupation labels *d′* was significantly better than those with low social status occupation labels, *d′*, *F* (1, 43) = 9.84, *p* = 0.01, *η*_*p*_^2^ = 0.419. Participants' discrimination for faces of different races did not differ significantly, *F* (1, 43) = 0.83, *p* > 0.05, *η*_*p*_^2^ = 0.026. The interaction between race and social status was significant, *F* (1, 43) = 4.86, *p* < 0.05, *η*_*p*_^2^ = 0.095. A subsequent simple effects analysis of the interaction found that when faces were native faces, participants' discriminatory index for faces with high social status occupations *d '* was significantly higher than the discriminatory indicator *d′* for faces with low social status occupations, *F* (1, 43) = 7.56, *p* < 0.05, *η*_*p*_^2^ = 0.496. When racial faces were other-race faces, participants' discriminatory index *d′* for faces with high social status occupations and the discriminative index *d′* for faces with low social status occupations were not significantly different, *F* (1, 43) = 1.116, *p* = 0.297, *η*_*p*_^2^ = 0.025. For high social status faces, the discrimination indicator *d′* of participants to recognize their own-race faces was significantly higher than that to recognize faces of other races, *F* (1, 43) = 6.73, *p* < 0.01, *η*_*p*_^2^ = 0.31; for low social status faces, there were no significant differences among races, *F* (1, 43) = 1.76, *p* > 0.05, *η*_*p*_^2^ = 0.023. The above results indicate that participants showed high recognition advantages for high social status faces when recognizing own-race faces, especially for high social statuses. However, they did not show the recognition advantages for high social status faces when recognizing other-race faces with different social statuses.

**Table 2:** Average hit rate, misrepresentation rate, d′, and C in Study 2.

Category	High social status				Low social status			
	Hit rate	False alarms rate	*d′*	*C*	Hit rate	False alarms rate	*d′*	*C*
Other-race	0.58 (0.36)	0.17 (0.64)	0.41 (0.61)	0.21 (0.12)	0.54 (0.21)	0.21 (0.63)	0.33 (0.57)	0.31 (1.02)
Own-race	0.73 (0.41)	0.21 (0.61)	0.52 (0.47)	0.12 (0.23)	0.24 (0.42)	0.12 (0.46)	0.11 (0.52)	0.34 (1.12)

The numbers outside the brackets in the table are the means, and the numbers inside the brackets are the standard deviations.

Reporting Criteria *C*: Participants' reporting criteria for faces of different social status did not differ significantly, *F* (1, 43) = 1.04, *p* = 0.313, *η*_*p*_^2^ = 0.024. Participants' reporting criteria for pictures of faces of different races did not differ significantly, *F* (1, 43) = 1.478, *p* = 0.231, *η*_*p*_^2^ = 0.033. The interaction between racial faces and social status was significant, *F* (1, 43) = 16.174, *p* < 0.001, *η*_*p*_^2^ = 0.273. A subsequent simple effects analysis of the interaction found that participants reported significantly higher criteria *C* for high social status faces when racial face was other-race face, than for low social status faces, *F* (1, 43) = 16.01, *p* < 0.001, *η*_*p*_^2^ = 0.27. When the racial face was an own-race face, participants reported criteria *C* more leniently for high social status faces, than for faces with low social occupational status, *F* (1, 43) = 6.61, *p* = 0.013, *η*_*p*_^2^ = 0.13.

### Discussion

Study 2 focused on the impact of motivation on the ORE in the opposite direction by attenuating participants' perceptions of the social status of other-race faces and, in turn, attenuating participants' motivation to individualize the processing of other-race faces through the principles of paired presentation and priming paradigms. The results showed that participants had a reduced advantage in recognizing other-race faces with high social status, suggesting that attenuated motivation reduced participants' recognition performance in identifying high social status other-race faces. It was also found that individuals' recognition scores for high social status own-race faces were significantly higher and better than those for low social status own-race faces. The result suggests that the priming stimuli and paired presentation manipulations increased participants' motivation to individualize and process own-race faces, further confirming motivation's important role in the ORE.

## General discussion

The current study found that perceivers' lack of motivation toward individualizing the processing of other-race faces was one of the main reasons for the ORE. That is, participants' recognition processes of other-race faces were influenced by motivation, both by increasing participants' motivation to individualize other-race faces (Study 1) and from the perspective of attenuating participants' motivation to individualize other-race faces (Study 2). The current study further validated Hugenberg et al.'s findings with social status as the operational variable and supported the CIM^12^.

The CIM suggests that more contact experience with one's own racial group members does not automatically moderate the ORE (Study 1). The results of the present study are not consistent with the contact experience theory, where individuals who have been in contact with members of their own racial group for a long time develop more perceptual experience in distinguishing and recognizing faces of their own racial group, resulting in recognition advantages for own-race faces. Furthermore, the theory suggests that if there is only contact with members of one's own racial group, there is no motivation to individualize the processing of other-race faces (Study 2) and it does not impact the ORE. This result is consistent with Young et al.^23^.

The increased performance in recognition of other-race faces with high social status may be because other-race faces with high social status satisfy the individual's need to be respected and admired by others, which in turn triggers a motivation for participants to individualize the processing of other-race faces. This is consistent with the CIM. The theory suggests that an other-race face that is relevant to the perceiver and satisfies some need of the perceiver is likely to motivate the perceiver to individualize processing. The reduced performance of participants in Study 2 in recognizing other-race faces with high social status may be due to the weakened motivation of participants to individualize the processing of other-race faces with high social status. This is possible because the presentation of scene pictures from a Chinese context during the experiment-initiated participants' perception of the social status of their own faces and reduced their perceptions of the social status of other-race faces. This did not satisfy the needs of individuals when they are racial identities, thus leading to lower motivation, which is consistent with Becker and Leinenger's study, where they found that a higher congruence of the priming stimulus with the face picture led to more attention and allocation of cognitive resources to congruent faces by the participants^30^.

The re-perception scores of high social status faces are significantly higher than those of low social status faces because high social status increases individuals' expectations of being respected, admired, and obeyed by others in their social environment, which leads to a strong motivation in individuals to pursue higher social status. Thus, individuals increase their feelings of being respected and obeyed by others by changing their behaviors to improve or maintain their social status from being threatened. For example, they were choosing jobs with a higher social status, making friends of higher social status, volunteering to take on more tasks at work, behaving positively, being more generous to others, and trying to maintain their public image^32,33^. There is a need to validate further the relationship between social status and social motivation through eye-movement techniques, EEG, and other brain imaging techniques, to gain more insight into the mechanisms underlying social status that influence individual behaviors.

However, there are limitations of the current study. Our study was only based on signal detection theory and did not measure response time, which may be a potential variable worth further exploration in the future. Because response time is correlated with the difficulty with which individuals perform the task of distinguishing between signal and noise, this is not as straightforward to understand in the framework of signal detection theory as it is in response time metrics^34^. It may provide new evidence to understand the influence of motivation in the ORE. In addition, due to the lack of cross-cultural comparisons in the current research, we cannot be obtained a direct effect of cross-culture. However, according to existing research, we speculate that cultural differences may also be a potential factor affecting the current research results. Future research should pay more attention to the potential role of cross-culture. It is worthy of noting that participants' social status might be an influential factor in shaping the ORE, which was not measured in the current study. This could be investigated in future studies.

Previous research has shown that the ORE exists not only across races^35^ but also between groups that can be visually distinguished (e.g., age, gender, religion) and even arbitrarily differentiated groups (e.g., universities and corporations)^16,36,37^. These ways of group differentiation were involved in various scenarios in everyday life. Future research could examine the relationship between motivation and ORE across groups of different natures to more comprehensively explore the causes of the ORE and attempt to reduce prejudice among different groups. All participants in this study were from China, but studies have suggested that there may be differences in individuals' individualized experiences and individualized motivations among different cultures, which in turn may produce different cognitive processing mechanisms for face recognition^38^. Therefore, it is necessary to include cultural differences in the independent variables for further investigation.

## Conclusion

Motivation plays an essential role in the generation of ORE. When increasing an individual's motivation to process other-race faces, the individual's recognition performance of other-race faces with high social status improves significantly, which attenuates the ORE. However, this recognition advantage disappears as motivation attenuates.

## Data Availability

All participants signed an informed consent form agreeing to participate in the study and the publication of the results and identifiable images. The datasets used and/or analysed during the current study are available from the corresponding author on reasonable request.
